# BioTM buzz (volume 5, issue 2)

**DOI:** 10.1002/btm2.10164

**Published:** 2020-05-15

**Authors:** Aaron C. Anselmo

**Affiliations:** ^1^ Division of Pharmacoengineering and Molecular Pharmaceutics, Eshelman School of Pharmacy University of North Carolina at Chapel Hill Chapel Hill North Carolina USA

## 1 DENDRIMER SIZE AND BRAIN TARGETING

Nanotechnologies have demonstrated considerable success in targeting specific tissues and breaching biological barriers for the treatment of various cancers. As such, improving nanoparticle performance through optimization of their physicochemical properties represents one of the most widely investigated topics in preclinical nanotechnology research. In this issue of Bioengineering & Translational Medicine, a team led by Professor Rangaramanujam Kannan from the Center for Nanomedicine, Wilmer Eye Institute at Johns Hopkins School of Medicine, describes a systematic study to determine how dendrimer size affects targeting of brain tumors in vivo. This work utilized two distinct orthotropic and clinically relevant brain tumor models in both mice and rats to show that Generation 6 dendrimers exhibited ~10‐fold higher tumor accumulation at 24 hr as compared to Generation 4 dendrimers. Generation 6 dendrimers were experimentally determined to be ~6.7 nm in diameter whereas Generation 4 dendrimers were ~ 4.3 nm in diameter, an important distinction that was most likely responsible for the significantly enhanced accumulation of the smaller Generation 4 dendrimers in the kidneys over a 48 hr period in both animal models. The authors associated this enhanced kidney accumulation with higher renal clearance and thus less persistence and shorter circulation in the vasculature, thereby limiting opportunities to interact with the brain tumors and decreasing accumulation. This work clearly demonstrates the importance of considering nanoparticle physical properties for their end application by highlighting how an ~2.3 nm difference in diameter could influence accumulation in brain tumors by ~10‐fold.^1^

## 2 STORAGE SYSTEMS FOR PHAGE THERAPIES

Phage therapies have recently received considerable interest, in part due to their potential in providing novel and complementary therapeutic mechanisms (as compared to antibiotics) for the treatment of various pathogenic infections. Phages are dominantly protein‐based and thus face many of the stability and formulation challenges of biologics. Researchers from the lab of Professor Hak‐Kim Chan from the Advanced Drug Delivery Group in the School of Pharmacy at the University of Sydney have developed a spray dried powder formulation of Pseudomonas phage PEV20 that maintains high activity after 250 days of storage. Through the use of plaque assays to evaluate phage titer over time under various temperature and relative humidity conditions and differential scanning colorimetry to determine the glass transition temperature of spray dried formulations, the authors concluded that as the glass transition temperature of the powders approached the storage temperature that the viable storage of phage decreased. Although these results may have been expected, this work highlights the need to develop and validate technologies for the implementation of phage‐based technologies.^2^

## 3 RED BLOOD CELL INSPIRED NANOPARTICLES FOR TOXIN REMOVAL

Recent work from Professor Jordan Green's lab in the Department of Biomedical Engineering at Johns Hopkins University details a nanotechnology capable of enhanced vascular circulation that was applied as a technology to facilitate detoxification in a sepsis model. The author's utilize red blood cell membranes and anisotropic shaped nanoparticles, thereby combining chemical and physical modifications, to improve detoxification as compared to nanoparticles with only a singular modification. Overall, this work highlights the importance of investigating and optimizing both the physical and chemical features of nanoparticles. Moreover, it was demonstrated that physical and chemical modifications to nanoparticles can act synergistically, thereby improving nanoparticle performance beyond what a single modification could afford.^3^
